# Development of Patient-Derived Human Monoclonal Antibodies Against Nucleocapsid Protein of Severe Acute Respiratory Syndrome Coronavirus 2 for Coronavirus Disease 2019 Diagnosis

**DOI:** 10.3389/fimmu.2020.595970

**Published:** 2020-11-13

**Authors:** Li Zhang, Binyang Zheng, Xingsu Gao, Libo Zhang, Hongxin Pan, Yong Qiao, Guangli Suo, Fengcai Zhu

**Affiliations:** ^1^ National Health Commission of the People’s Republic of China, Key Laboratory of Enteric Pathogenic Microbiology, Jiangsu Provincial Center for Disease Control and Prevention, Nanjing, China; ^2^ Department of Laboratory, Nanjing Red Cross Blood Center, Nanjing, China; ^3^ CAS Key Laboratory of Nano-Bio Interface, Suzhou Institute of Nano-Tech and Nano-Bionics, Chinese Academy of Sciences, Suzhou, China

**Keywords:** SARS-CoV-2, nucleocapsid protein, human antibody, phage display, sandwich ELISA

## Abstract

The pandemic caused by emerging Severe Acute Respiratory Syndrome Coronavirus 2 (SARS-CoV-2) presents a global public health threat. Illustrating human antibody responding to viral antigen could potentially provide valuable information for basic research and clinical diagnosis. The antibody can be used as a complement to the viral detection for the rapid diagnosis of infected patients. Compared with spike protein (SP), nucleocapsid protein (NP) is normally conserved and highly immunogenic in many coronavirus members. As a major antigen, NP is a potential target for the diagnosis of SARS-CoV-2 infection. Here, we constructed a combinatorial fragment of antigen-binding (Fab)antibody phage library based on peripheral blood-derived from five coronavirus disease 2019 (COVID-19) infected donors. From the library, 159 Fab antibodies were obtained and identified by panning with NP. Among them, 16 antibodies were evaluated for their binding properties and epitopes recognition. Among these 16 antibodies, two well-paired antibodies were finally screened out for SARS-CoV-2 diagnosis by double-antibody sandwich enzyme-linked immunosorbent assay (ELISA) method. Our works may provide a potential resource for the clinical diagnosis of SARS-CoV-2 infection.

## Introduction

The COVID-19 pandemic caused by severe acute respiratory syndrome coronavirus 2 (SARS-CoV-2) has spread across the globe rapidly and results in an unprecedented public health crisis (1–3). The COVID-19 symptoms have reportedly ranged from asymptomatic infection to severe acute respiratory syndrome that can ultimately lead to death (4, 5). Due to the rapid deterioration of infections, implementing fast and universal diagnostic tests is predominant to curb the pandemic. But the true extent of the spread of SARS-CoV-2 is not fully be realized maybe because of limited testing (6).

Like other viral tests, viral nucleic acids and viral-specific proteins can be used to detect viral infection in patients to diagnose viral infection. Reverse transcription polymerase chain reaction (RT-PCR) was the first method developed for COVID-19 diagnosis and is the current gold standard (7). However, the nucleic acid based diagnose has some obvious defects. Firstly, the quality of RT-PCR depends greatly on the quality of the throat swab and the proficiency of the experimenter. Secondly, it will take several hours and require specialized reagents and instruments to perform RT-PCR. Thirdly, many viral RNA is instability and susceptible to degradation during transport and storage. However, the detection of viral-specific protein has the same effect as that of nucleic acids. Moreover, the proteins are more stable than RNA, and point-of-care detection reagent could be utilized in protein detection.

To establish a high-quality antigen detection method, we should understand the etiology of COVID-19 and chose the candidate antigen for further research. SARS-CoV-2 is an enveloped, positive-RNA virus which belongs to lineage B of the beta-coronavirus family, and shares about 80% identity with severe acute respiratory syndrome coronavirus (SARS-CoV) (8, 9). Like other coronaviruses, the SARS-CoV-2 genome encodes four structural proteins including spike (S), envelope (E), membrane (M), and nucleocapsid (N) protein. The nucleocapsid protein (NP) of 419 amino acids protects the viral RNA and acts as a scaffold for viral packaging (10–12). The NP of SARS-CoV-2 could self-assemble (13) and provide a RNA binding domain in the virus life cycle (14). The primary function of the N protein is binding to the viral RNA genome and packing them into ribonucleoprotein (RNP) complex (15).

It has been reported that viral NP of SARS-CoV-2 is normally very conserved with strong immunogenicity and is lavishly expressed during infection (16), suggesting that NP can be potentially used for early diagnosis of SARS-CoV (17–19) and MERS infection (20). It is reported that antibodies to the NP of SARS-CoV-2 are more sensitive than the spike protein (SP) antibodies for early infection detection (21, 22). Furthermore, immunoglobulin M (IgM) and immunoglobulin G (IgG) antibodies against NP have been successfully detected in serological testing and epidemiological monitoring (23, 24). Therefore, humoral immune response characteristics against NP and related specific monoclonal antibodies need further investigation. In this study, we constructed a comprehensive human antibody library derived from five COVID-19 patients in the convalescence period using phage display technology for the first time. A panel of human monoclonal antibodies was obtained by panning with purified NP. Subsequently, the character of antibodies, including binding specificity, affinity to NP, targeted antigenic epitopes, and application in diagnostic was evaluated. This study facilitates the understanding of humoral responses of SARS-CoV-2 infection and helps to develop new diagnostic tools for detecting and diagnosing viral antigen at various infection stages, which could be ultimately applied in clinical work as well as in epidemic surveillance.

## Materials and Methods

### Study Approval

The informed consent from 5 donors was obtained for blood and cell samples, followed by the approval from the Institutional Review Board of the Jiangsu Provincial Center of Disease Control and Prevention. This study was reviewed and approved by the Ethics Committee of Jiangsu Provincial Center for Disease Prevention and Control, Nanjing, China.

### Preparation of Blood Samples From Convalescent Human Donors

Peripheral blood samples were collected from five COVID-19 convalescing patients admitted in Huai'an No 4 People's Hospital of Jiangsu province, China. Peripheral blood mononuclear cells (PBMCs) were isolated using Ficoll-Paque Plus (GE Healthcare, Sweden) density gradient media according to the manufacturer’s protocol. Briefly, blood samples were diluted with the same volume of phosphate-buffered saline (PBS) (Gibco, USA) and slowly transferred over Ficoll-Paque in the SepMate-50 tube (Stemcell, Canada). After horizontal centrifugation at room temperature at 800 g for 20 min, PBMCs were collected and transferred into a new centrifuge tube. Following two steps of washing with PBS, the total RNA of PBMCs was extracted using the RNeasy Mini Kit (Qiagen, Germany) following the manufacturer’s instructions.

### Preparation of Recombinant NP

The SARS-CoV-2 gene (GenBank: MT066176.1) with 1,260 bp was synthesized and cloned into the prokaryotic expression vector pET28a, and then expressed and purified as previously described (25).

### Construction of the Phage Antibody Library Pool

The five RNA samples extracted from the PBMCs derived from five COVID-19 convalescing patients were used as templates for cDNA synthesis by Transcriptor High Fidelity cDNA Synthesis kit (Roche, Germany). Five cDNA samples were mixed equally to form a pool of templates. Full-length light chain genes and Fd fragments (a variable region and a CH1 domain of a heavy chain) of heavy chain genes were amplified from the cDNA template pool by PCR using the primer pairs 5VK, 7VL, and 8VH gene family as described elsewhere (26). Light chain genes were cloned into the phagemid vector pComb3H between *Xab I* and *SacI* restriction sites to form the light chain gene pool. The heavy chain genes were sequentially cloned into a light chain pool in pComb3H between *Xho I* and *Spe I* restriction sites following a standard protocol published previously (27). By two-step cloning, the final anti-SARS-CoV-2 phage antibody library pool was constructed. The phage antibody library pool was aliquoted and frozen at -80°C until further use.

### Panning Phage Antibody Library With NP

Recombinant SARS-CoV-2 NP expressed in *E. coli* was used for panning the phage antibody library pool following the procedure described previously (28). Briefly, NP was coated in the immune tubes and incubated at 4°C overnight. After washing by Phosphate-buffered saline with 0.05% Tween20 (PBST), the phage antibody library was added, and the tube was incubated at 37°C for 2 h, followed by removal of the supernatant and washing with TBST for five times. Finally, the enriched phage antibody library pool was eluted using 0.1M Glycine-HCl (pH 2.2). With the same procedure, the second and third rounds of panning for screening specific binding antibodies were performed by phage display. After three rounds of panning, single clones specific to SARS-CoV-2 NP were picked for later use.

### ELISA for Screening the Specific Binding Antibodies

After three rounds of panning, individual clones from the enriched phage pool were evaluated for the binding specificity to NP. Briefly, 96-well enzyme-linked immunosorbent assay (ELISA) plates were coated with purified NP at 4°C overnight. After blocking and washing, single clones from the enriched pool were added, and four blank control wells were performed simultaneously. Following five times wash with PBST, horseradish peroxidase (HRP) conjugated anti-human Fab (Sigma Aldrich, USA) was added and incubated at 37°C for 1 h. After washing with PBST, 3,3’,5,5’-tetramethylbenzidine substrate (Thermo Scientific, USA) was added and incubated at room temperature for 10 min, following the color change was monitored at 450 nm by adding 50 μL 2M H_2_SO4 to stop the reaction. The cut-off value was set as the mean of the blank controls plus 2 fold of standard deviation (X+2SD). The value of OD 450 higher than the cut-off value was considered as positive. In this way, single phage antibodies against NP were obtained for further use.

### Sequencing and Genetic Analyses of Antibodies

All the single phage antibodies binding to NP were sequenced in the Sangon biotech company (www.sangon.com). Antibody sequences were run through the blast and aligned with homologous sequences of the IMGT (www.imgt.org/) database. The phylogenetic tree of variable regions was constructed by MEGA 6.0 software. The full length of variable region amino acids was aligned using the web servers of ESPrit 3.0 (http://espript.ibcp.fr/ESPript/ESPript/). The sequence logos of the Heavy chain, Lambda chain, and Kappa chain were generated using the web server of Weblogo (http://weblogo.berkeley.edu/logo.cgi). Based on the diversity of variable genes by primary sequence alignments and the distance in the phylogenetic tree and OD values of indirect ELISA the specific candidate antibodies plasmids were optimized.

### Expression and Purification of Antibodies

Heavy and light chain genes of optimized antibody plasmids were cloned into the PTT5 vector containing the constant regions of human IgG1, respectively. Eukaryotic expression vectors of heavy and light chains were co-transfected into HEK-293F cells by Polyetherimide (PEI, Polysciences). In brief, 500 μg of heavy chain expression plasmids and 500 μg of light chain expression plasmids were mixed with 3.0 mL PEI (1 mg/mL) and transfected into 1 L HEK-293F cell culture at a density of 1.5 × 10^6^ cells/mL. After transfection, HEK-293F cells were kept shaking at 37°C with 8% CO_2_ for antibody expression. After 7 days, the culture supernatant was harvested by centrifuging at 10,000 g for 10 min and filtered through a 0.22 μm membrane. Protein A column (GE Healthcare, Sweden) was used for antibody purification by NGC Quest 10 Plus system (Bio-Rad, USA). Concentrations were determined by BCA Protein Assay Kits (Thermo Scientific, USA). The purified antibodies were aliquoted and frozen at -80°C until further use.

### Indirect ELISA Assay

Purified NP was coated onto 96-well plates in 1μg/ml and incubated at 4°C overnight. Plates were blocked with 1% bovine serum albumin (BSA) at 37°C for 1 h. Purified anti-NP IgG antibodies (JS01–JS16) were prepared in concentration of 1mg/ml and serially diluted from 1:10,000 to 1:1 280,000. All 16 serially diluted antibodies were added into plates and incubated at 37°C for 1 h. HRP-conjugated goat anti-human Fc antibody (Sigma Aldrich, USA) was used as a secondary antibody at 37°C for 1 h. 3,3’,5,5’-tetramethylbenzidine substrate (Thermo, USA) was used, and the absorbance at 450 nm was measured by a plate reader (Tecan, Switzerland).

### Western Blot Analysis

Purified SARS-CoV-2 NP was electrophoresed by 1 μg/well in NuPAGE 4–12% Bis-Tris gradient gel and transferred to nitrocellulose membrane (GE Healthcare, USA). Membranes were blocked by 1% BSA for 1 h at room temperature; the membrane-immobilized proteins were probed by purified human antibodies (20 mg/ml) overnight at 4°C. Monoclonal goat anti-human antibody (Sigma Aldrich, USA) was used as the secondary antibody. Bands were visualized by 3, 3’ diaminobenzidine (DAB) according to the manufacturer’s instructions.

### SPR Analysis

The interaction between purified antibodies and SARS-CoV-2 NP was performed by SPR using the BIAcore T200 system (GE Healthcare, USA) carried out at 25°C in single-cycle mode. Purified SARS-CoV-2 NP diluted in 10 mM sodium acetate buffer (PH 5.5) was immobilized to CM5 sensor chip by amine coupling reaction. Serially diluted antibodies were injected with a rate of 30 μl/min in sequence. The equilibrium dissociation constants (binding affinity, Kd) for each antibody were calculated using Biacore T200 Evaluation Software.

### Antibody-Sandwich ELISA

To screen antibodies for the detection of NP, 16 antibodies were used to establish a sandwich ELISA. JS08 was labeled with HRP at 1 mg/mL according to manufacture instruction. The best suitable concentration of coating antibody and detection antibody were determined by the checkerboard. The sandwich ELISA was performed as follows. 16 antibodies were coated onto 96 well plates at a concentration of 1μg/mL in carbonate bicarbonate buffer at 4°C overnight. After blocking with PBS containing 1% BSA, 100 μL serially diluted samples were added and incubated at 37°C for 1 h. After rinsing, HRP-conjugated JS08 was added. The reaction was developed by tetramethylbenzidine (TMB) substrate solution and the absorbance at 450 nm was measured by a plate reader (Tecan, USA).

### Competition ELISA

NP based competition ELISA was used to categorize the epitope of the 16 antibodies. Briefly, SARS-CoV-2 NP was onto microplates at 2 μg/mL at 4°C overnight. The first antibody was serially diluted from 5 μg/mL to 0.04 μg/mL and incubated at 37°C for 1 h. The same volume of PBS was operated the same as the first antibody and used as control. After washing, HRP-conjugated secondary antibody was added at 1:1,000 dilution. By incubation for 30 min, OD450, the value was measured. Competition tolerance was calculated by the percentage decrease to compare to PBS well. The competition percentage was calculated as follows:

$$Competition\;percentage=\frac{Value\;of\;PBS\;well-Value\;of\;first\;antibody\;well\;}{Value\;of\;PBS\;well}\times 100\%$$

### Statistical Analysis

Statistical significance was determined by unpaired two-tailed student’s *t*-test and one-way analysis of variance (ANOVA). P < 0.05 was considered statistically significant. The average or mean data were calculated using Microsoft Excel or GraphPad Prism 6.07 software.

## Results

### Generation of Human Antibodies Against NP of SARS-CoV-2

**Figure 1:**
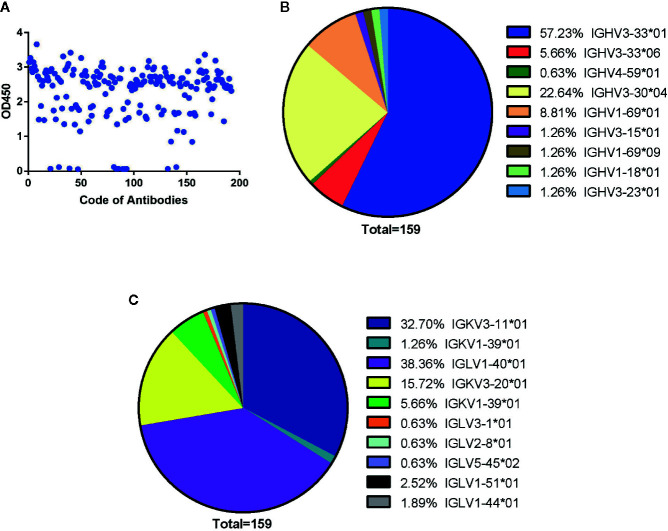
Generation of human antibodies against SARS-CoV-2 NP by screening phage libraries. **(A)** 192 randomly picked colonies were screened by ELISA to evaluate binding activity to SARS-CoV-2 NP. The data are expressed as an average of triplicate measurement of absorbance at 450 nm. All single phage antibodies binding to NP were sequenced, and variable gene sequences of heavy and light chains were run through the blast and aligned with homologous sequences of the IMGT (www.imgt.org/) database. The germline distribution of heavy **(B)** and light **(C)** chains were depicted in the pie chart.

Two independent anti-SARS-CoV-2 Fab fragment antibody libraries, one for lambda chain and one for kappa chain, was established using the pComb 3H vector system. The full length of kappa and lambda light chain (VL and CL1 domain), and Fd fragment of the heavy chain (VH and CH1 domain), were derived from five COVID-19 patients from Huai’an city in Jiangsu province. These two phage libraries contained 1 × 10^9^ and 1.4 × 10^9^ independent clones and 100% Fab genes diversity, which was confirmed by sequencing. After three rounds of panning, 192 randomly picked colonies were further screened by ELISA to evaluate binding activity to SARS-CoV-2 NP. 178 positive clones were finally identified (**Figure 1A**).

### Sequence Alignment Analysis for Generated Antibodies

By sequencing all positive clones, 159 paired heavy and light chain clones were obtained and aligned by ClustalW methods of MEGA software (Version 6.0). FastTree 2.3 was used to construct the phylogenetic tree (**Figures S1** and **S2**). According to the phylogenetic tree and DNAPLOT program of the VBASEA database (www.vbase2.org), the germline and gene families of these 159 isolated Fab antibodies were identified. The IGHV3-33*01 and IGHV3-30*04 were predominant subtypes accounting for 57.23 and 22.64% respectively, while other subtypes of the IGHV3 family accounted for 8.18% of the total number. Three subtypes of the IGHV1 family (IGHV1-69*01, IGHV1-69*09, IGHV1-18*01) constituted approximately 8.81, 1.26, and 1.26% of VH repertoire respectively. IGHV4-59*01 was the lowest germline, with only 0.63% of the VH repertoire (**Figure 1B**).

For the light chain, the distribution of gene repertoire was more dispersed than VH genes. Kappa light chain accounting for 55.35% consisted of three subtypes: IGKV3-11*01 (32.70%), IGKV3-20*01 (15.72%), and IGKV1-39*01 (6.95%). Lambda light chains with 44.66% proportion were derived from six germline subtypes: IGLV1-40*01 (38.36%), IGLV3-1*01 (0.63%), IGLV2-8*01 (0.63%), IGLV5-45*02 (0.63%), IGLV1-51*01 (2.52%), and IGLV1-44*01 (1.89%) (**Figure 1C**). From these results, we found that two isotypes of light chains were nearly equally distributed with dominant subtypes: IGKV3-11*01(32.70%) in the kappa chain and IGLV1-40*01(38.36%) in the lambda chain (**Figure 1C**).

**Figure 2:**
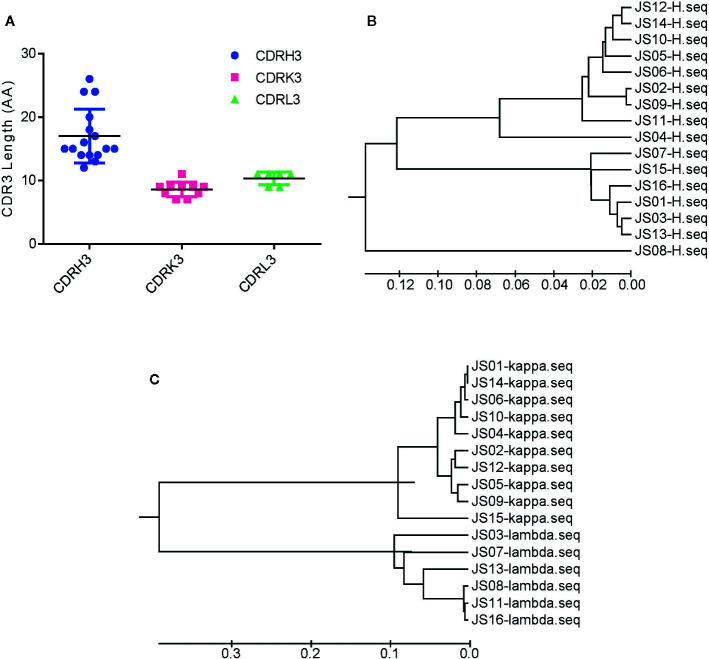
Sequences analysis of SARS-CoV-2 NP-specific antibodies. **(A)** The number of amino acids of CDR3s. CDRH3, CDRK3 and CDRL3 were shown respectively. The error bar means the mean ± standard deviation (SD). Phylogenetic tree of VH **(B)**, Vκ, and Vλ **(C)** constructed by MEGA 6. The bars indicate the distance scales in trees measured by amino acid differences of variable regions.

Based on the diversity of variable genes by sequencing alignments and the distance in the phylogenetic tree and OD values of indirect ELISA, there are 159 strains of antibodies, 16 unique clones named from JS01 to JS16 were selected out as candidates for further interrogation. Amino acid sequences of the full length of variable chains and complementarity-determining regions (CDRs) were aligned by using the web servers of ESPript 3.0 (http://espript.ibcp.fr/ESPript/ESPript/) (**Figures S3** and **S4**). The sequence logos of the Heavy chain, Lambda chain and Kappa chain were generated using the web server of Weblogo (http://weblogo.berkeley.edu/logo.cgi) (**Figure S5**). The average CDR3 lengths of VH, VK, and VL were 17, 8.6, and 10.3 amino acids. However, the CDR3s of heavy chains had more diversity than light chains (**Figure 2A**). The large difference of variable regions might result from a high frequency of somatic hypermutation and affinity maturation during the humoral immune response. From phylogenetic trees by sequences of 16 antibodies, antibodies with kappa light chains account for 10/16 and those with lambda light chains account for 6/16, while antibodies with the heavy chain of IGHV1 and IGHV3 are proportionally similar (**Figures 2B, C**). Notably, the variable region of the JS08 antibody has a significant variation compared with other antibodies, according to the distance from the VH phylogenetic tree (**Figure 2B**).

### Confirmation of Candidate IgG Antibodies and Binding Properties

**Figure 3:**
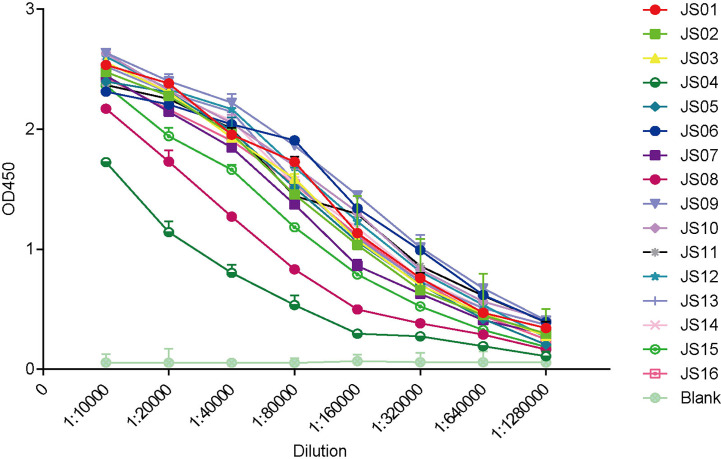
ELISA binding curves of 16 antibodies against SARS-CoV-2 NP. The horizontal axis shows different dilutions of SARS-CoV-2 NP-specific antibodies. The different color lines mean 16 candidate antibodies (JS01-16). “Blank” means blank control of 5% skimmed milk. The data are expressed as the average of triplicate measurement of absorbance at 450 nm. The error bar means a mean ± standard deviation (SD).

**Figure 4:**
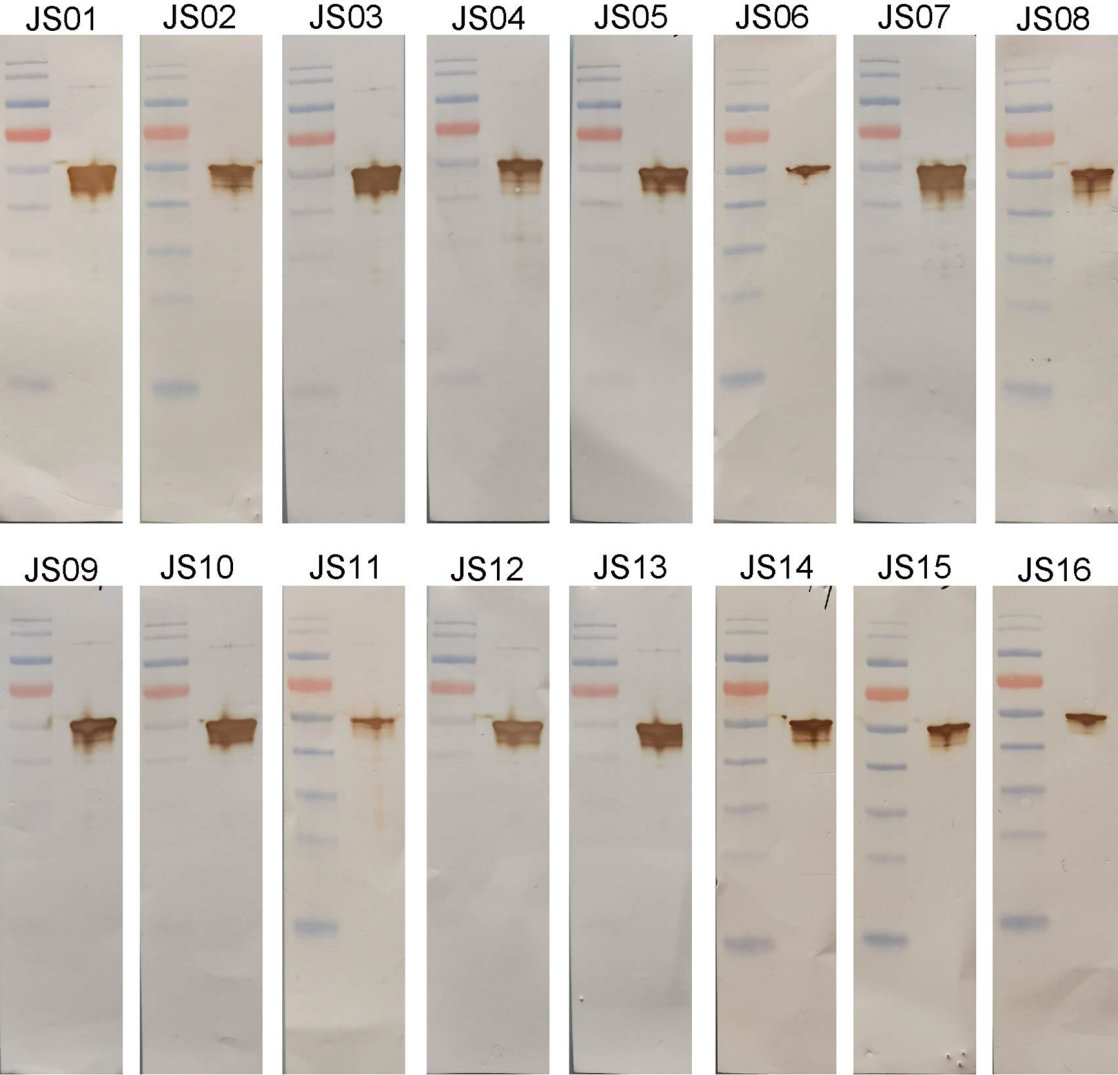
Western blot analysis of antibodies using purified NP. SARS-CoV-2 NP was electrophoresed and transferred to membranes and then probed by human antibodies and secondary antibodies and visualized by DAB.

The full-length of human IgG1 antibody was purified and prepared in 1mg/mL for later use. Indirect ELISA and Western Blot were performed to certify the binding specificity between 16 strains of antibodies and COVID-19 NP. For the indirect ELISA assay, coated SARS-CoV-2 NP was recognized by 16 human antibodies in turn and detected by HRP-conjugated anti-human Fc as the secondary antibody. The cut-off value was 0.383 and the antibody titers were ranged from 1:80 000 to 1:1280 000 (**Figure 3**). All antibodies were further evaluated by Western Blot analysis with SARS-CoV-2 NP. As shown in **Figure 4**, a band with a size of 50 kDa was detected in all 16 candidate antibodies, which suggested that all candidate antibodies could bind SARS-CoV-2 NP effectively with linear epitopes (**Figure 4**).

### All Candidate Antibodies Have a High Affinity to Bind SARS-CoV-2 NP

**Figure 5:**
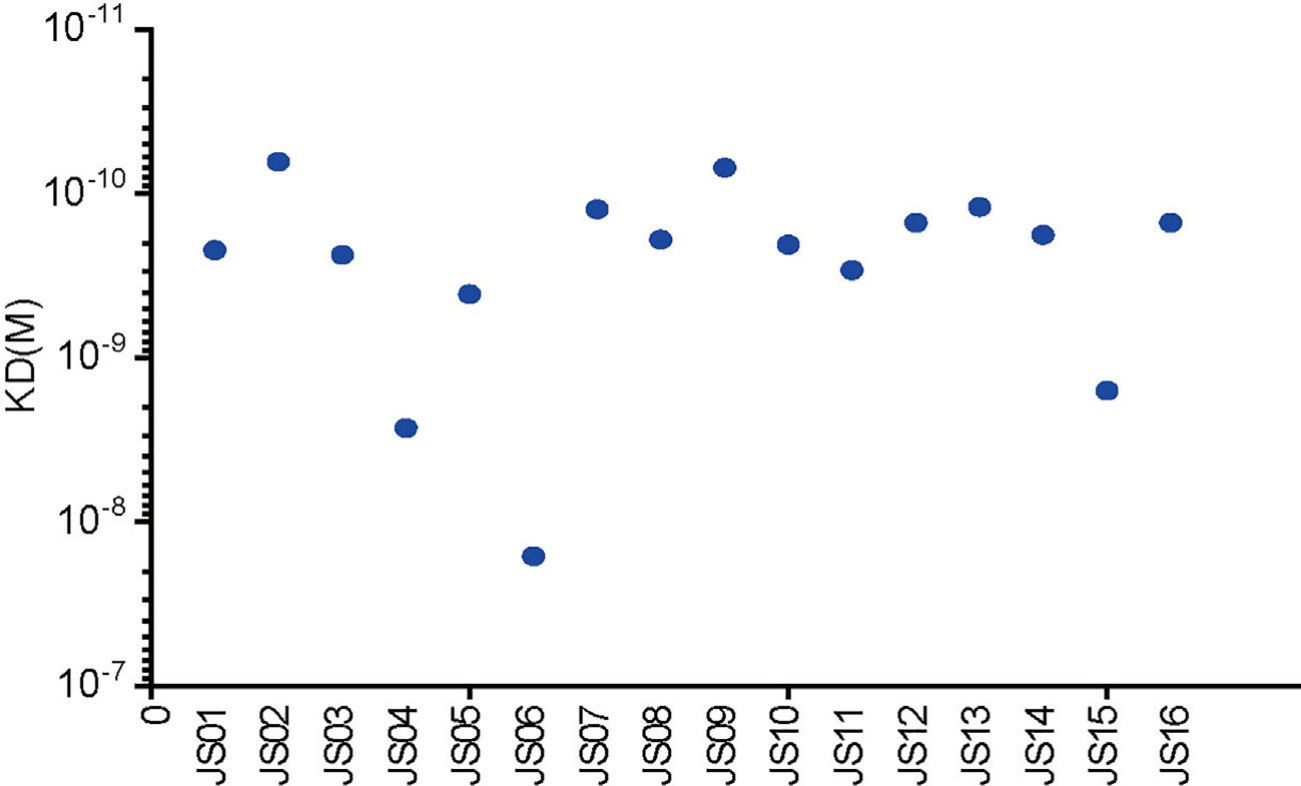
Affinity analysis of the binding of 16 antibodies to SARS-CoV-2. The kinetic rate constants of 16 antibodies and SARS-CoV-2 NP were evaluated by the BIAcore T200 system.

Surface plasmon resonance (SPR) assay was performed to evaluate binding specificity and affinity between antibodies and purified NP. Our results showed that all candidate antibodies exhibit a tight binding affinity to immobilized NP. The KD values of all 16 candidate antibodies range from 16 nM to 0.069nM. Only three antibodies (JS04, JS06 and JS15) had nM level of KD values, while other antibodies were all with sub-nM levels of KD (**Figure 5**). All kinetic curves of 16 antibodies were recorded and depicted in **Figure S6**.

### Paired Antibodies Could Capture NP of SARS-CoV-2 by Sandwich ELISA

**Figure 6:**
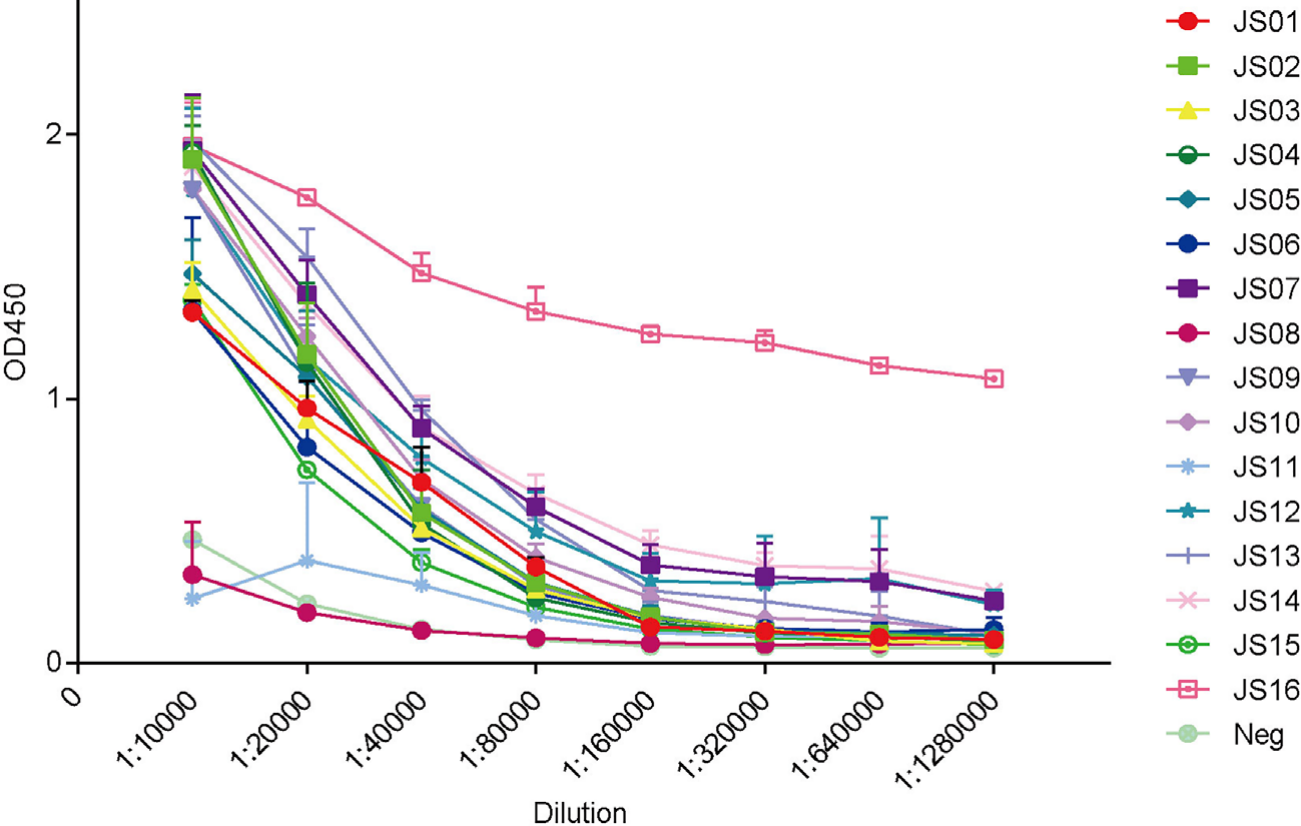
Evaluation of double antibodies against capture SARS-CoV-2 NP by ELISA. The horizontal axis shows different dilutions of SARS-CoV-2 NP-specific antibodies. The different color lines mean 16 candidate antibodies (JS01-16). The different color lines mean 16 candidate antibodies. “Neg” means unrelated antibody coated in the ELISA plate. The data are expressed as an average of triplicate measurement of absorbance at 450 nm. The error bar means a mean ± standard deviation (SD).

To establish antibody-sandwich ELISA to screen several antibody pairs. We first selected two antibodies, JS08 and JS12 with a maximum distance in the phylogenetic tree in **Figure 2B** to evaluate the optimal concentrations of the coated antibody and second antibody by checkerboard ELISA (**Figure S7**). Based on the result, the detection limit of SARS-CoV-2 NP was less than 3.9 ng/mL, while the concentration of coated antibody only had a certain impact on sensitivity. Therefore, we selected 2 μg/mL of the coated antibody for further interrogation. Antibody-sandwich ELISA was performed to scan the best pair between coated candidate antibodies and HRP-conjugated JS08. According to the ELISA result, we found that JS08 could not pair with JS06 and JS11, and the left 13 candidate antibodies could cooperate with JS08 to capture SARS-CoV-2 NP. Finally, we found that the JS16 was the best partner of JS08, with the optimal detection limit below 0.78ng/mL. However, their minimum detection values ranged from 12.5 ng/mL to 1.56 ng/mL (**Figure 6**).

### The Candidate Antibodies Recognize Different Epitopes of NP

**Figure 7:**
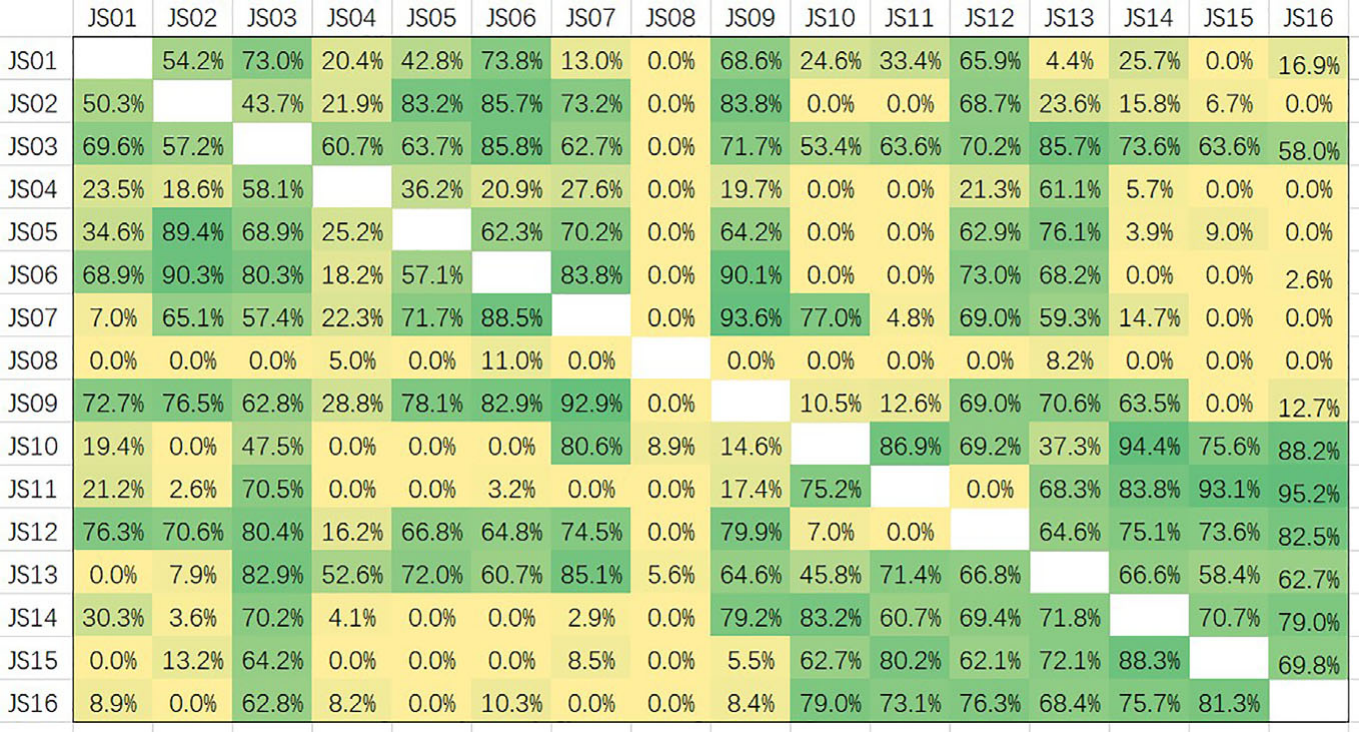
Competition assays and epitope binning of 16 antibodies (JS01-16) by using competitive ELISA. The competition percentages were shown for each pair of antibodies. Values less than 20 indicate antibodies had noncompetitive epitopes; Values between 20 and 60 indicate intermediate binding sites; Values more than 60 represent antibodies shared overlapping or close epitopes.

Epitopes of all 16 candidate antibodies were analyzed by competition ELISA (**Figure 7**). Competitiveness is defined by the values of competition percentages calculated by the given formula. A paired antibodies are defined as non-competing if the value is less than 20%, are deemed to compete the same epitope if the value is greater than 60%, and are considered a part of overlapping if the value is between 20 and 60 (29). Based on the data of competition, all the candidate antibodies can be roughly divided into five groups. JS08 has no competition for all other candidates, indicating a unique epitope JS08 has; JS04 only has a partial competition with JS03 and JS13 belonging to another site; JS03 and JS13 have a nearly the same epitope. The left antibodies can be divided into two groups. One group contains JS01, JS02, JS05, JS06, JS07, and JS09. The other group includes JS10, JS11, JS14, JS15, and JS16. These two groups have no competition with the former three groups, but they intermediately competed against each other, suggesting these two groups of antibodies may have a close epitope.

## Discussion

COVID-19 can cause severe disease including fever, cough, shortness of breath and pneumonia. Some severe cases lead to a variety of complications and even death. As a newly emerged viral pathogen to the human immune system, there is currently no specific immunity in the population. Humans of all ages and races are susceptible to SARS-CoV-2 infection (30, 31). Recently, the main methods to curb this epidemic is to ensure an early and accurate diagnosis of the viral infection and appropriate quarantine (32, 33). The early diagnosis of COVID-19 is a key factor in the treatment and the control of the transmission.

Generally, RT-PCR is a broadly used classical method for COVID-19 diagnosis. However, the defects of this method are expensive, time-consuming, and inevitably false negative and false positive. Antigen detection assay which detects viruses, may make a beneficial supplement to the imperfect RNA-based tests. Spike protein of SARS-CoV-2 has been detected by sandwich enzyme-linked immunosorbent assay and thio-nicotinamide adenine dinucleotide cycling (34). However, this method just has a low limit of detection with 2.3 × 10^-18^ moles/assay. Another research evaluated a commercial rapid antigen test kit of SARS-CoV-2 and found that the antigen test kit was 10^3^ fold less sensitive than viral culture and 10^5^ fold less sensitive than RT-PCR (19). NP can be potentially used as a target for early diagnosis of SARS-CoV-2 for its conservativeness and strong immunogenicity. A recent study has reported several monoclonal antibodies against the NP of SARS-CoV-2 which might form the basis of a future rapid antigen detection test (35). So far, the efforts are far from enough. Higher qualified antibodies and more sensitive antigen detection methods are still urgently needed in COVID-19 diagnose.

Compared with SP, NP is more abundantly produced during infection, exhibits stronger immunogenicity, and is normally conserved, acting as an ideal antigen for viral antibody detection (22, 36). NP was reported as a good early diagnostic marker for severe acute respiratory syndrome (SARS) (37–39). Therefore, the development and characterization of antibodies against NP are very meaningful for COVID-19 diagnosis.

Recently, it has been reported that many human antibodies against SARS-CoV-2 are isolated by single B cell sorting (40–43) or from humanized monoclonal antibody (44). The advantage of single cell sorting is allowing direct acquirement of antibody genes containing a pair of heavy and a light chain from a single B cell. But this method is very dependent on the single-cell technologies about precise assay of antibody secretion and single-cell precise picking (45). So far, these technologies are generally time-consuming, low-throughput screening, and high-cost (46). Further, antibodies derived from a single B cell always have a low affinity to the target antigen. Phage display technology has emerged as a powerful tool for antibodies screening with the advantages of simplicity, high efficiency and low cost. Especially, microfluidic-based phage display enables screening with high throughput, high efficiency and high integration (47). In this study, the antibody cDNA library was constructed based on the total RNA isolated from a pool of PBMCs containing many B cells secreting antibodies including NP antibodies. Through the construction of a phage library using antibody an cDNA library, we finally screened out the antibodies against NP.

In this study, human antibodies against SARS-CoV-2 NP were generated by phage display technology. These antibodies were screened from a library containing more than 2.4 × 10^9^ independent clones. So different germlines and isotypes of antibodies with great diversity in sequences were obtained, and 16 unique antibodies were selected as a candidate for further interrogation. As antibodies were panned in an optimum and stringent condition from mass clones in a library, they usually had a high binding affinity to the target antigen, which was also verified by SPR assay, and the highest affinity in antibodies was 0.069 nM. Besides close affinity, antibodies also had a broad diversity. Based on the Phylogenetic tree, JS08 shown a great difference with other antibodies, indicating JS08 might have a unique character and have no overlapping epitopes with other antibodies. This conclusion was subsequently certified by competition ELISA and at least five epitopes in N protein were identified. We also developed a double-antibody sandwich ELISA to screen the well-paired antibodies for future use in COVID-19 diagnosis. Finally, we found that JS16 could make the best pair with JS08 with the detection limit lower than 0.78 ng/mL.

However, there are still some limitations to this study. Firstly, due to the strict restriction of biological safety for SARS-CoV2 experiments, the selected paired antibodies were only evaluated by recombinant NP expressed by *E. coli*, not by the live virus. Before it was used as a diagnostic reagent in the clinical, it has been evaluated by live viruses from different kinds of samples such as throat swab, bronchoalveolar lavage fluid, and patient serum. Secondly, the characteristic analysis of 16 candidate antibodies needs further in-depth investigation by more academic approaches. The definite binding sites and the structure of the antibody-NP complex. Thirdly, the PBMC samples derived from relatively small numbers of donors were used for NP antibody screening, although the constructed antibody library was large enough for us to screen out well-paired NP antibodies. If conditions permit, more samples derived from more donors should be adopted to expand the antibody diversity for more well-paired NP antibodies yield.

There are lots of works needed to be completed in the future. Sandwich ELISA based method is not an all-purpose for every application scenario. Point-of-care viral antigen detection is urgently needed because it is easy to be carried and operated. We plan to label nanoparticles or colored latex nanoparticles to JS08 or JS16 antibody to develop a rapid diagnostic test method for clinical use. These antibodies could also be used in virus fundamental research.

In conclusion, we generated and characterized a panel of human antibodies against NP of SARS-CoV-2. We found that 16 antibodies may recognize five epitopes on NP, which provides an insight into the humoral response induced by SARS-CoV-2 infection. Furthermore, two well-paired antibodies were screened out for COVID-19 diagnosis by double-antibody sandwich ELISA method. This work provides a potential tool for monitoring viral antigens in various clinical samples and guiding treatment for patients.

## Data Availability Statement

The sequence data regarding antibody sequences have already been uploaded successfully on GeneBank. The accession number for each sequence is from MW116179 to MW116210.

## Ethics Statement

The studies involving human participants were reviewed and approved by Ethics Committee of Jiangsu Provincial Center for Disease Prevention and Control, Nanjing, China. The patients/participants provided their written informed consent to participate in this study.

## Author Contributions

GS, FZ, and HP conceived, designed, and supervised the whole study. LZ, XG, and BZ contributed in construction of the antibody libraries, panning and sequencing of mAbs, and generation of mAbs. LBZ and YQ performed ELISA, Western Blot, and SPR assays. LZ and GS wrote the paper. All authors contributed to the article and approved the submitted version.

## Conflict of Interest

The authors declare that the research was conducted in the absence of any commercial or financial relationships that could be construed as a potential conflict of interest.
