# Supplementary material for: Development of Patient-Derived Human Monoclonal Antibodies Against Nucleocapsid Protein of Severe Acute Respiratory Syndrome Coronavirus 2 for Coronavirus Disease 2019 Diagnosis
Source: Front Immunol. 2020 Nov 13;11:595970. doi: 10.3389/fimmu.2020.595970 (PMC7691652; doi:10.3389/fimmu.2020.595970)
Supplement: Supplementary file 1 [file DataSheet_1.docx]

Figure S1


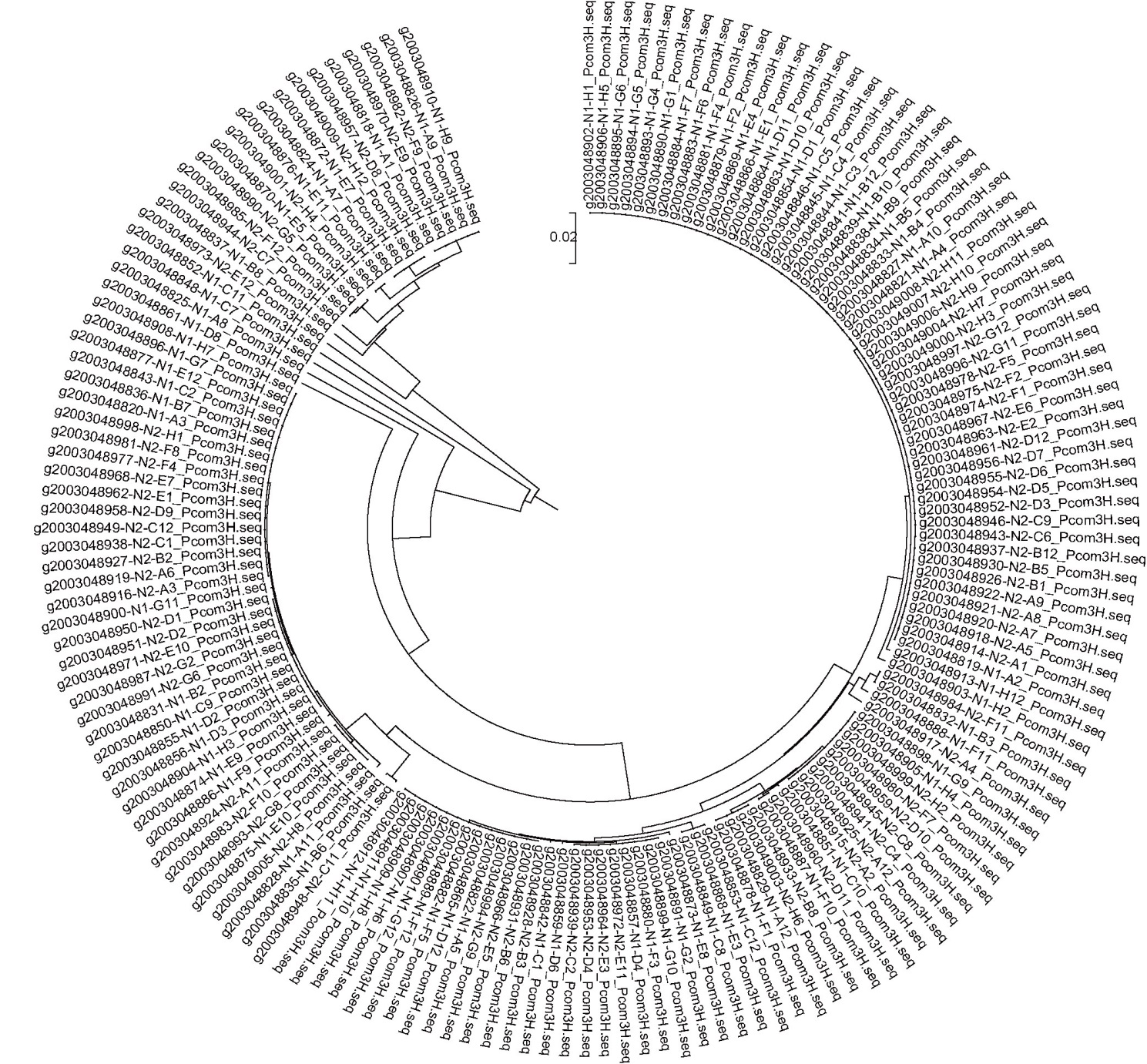


Figure S1. The circular form of the phylogenetic tree is based on variable regions sequences of heavy chains of selected Fabs. The phylogenetic trees were constructed by the minimum evolution method implemented of software MEGA6.

Figure S2


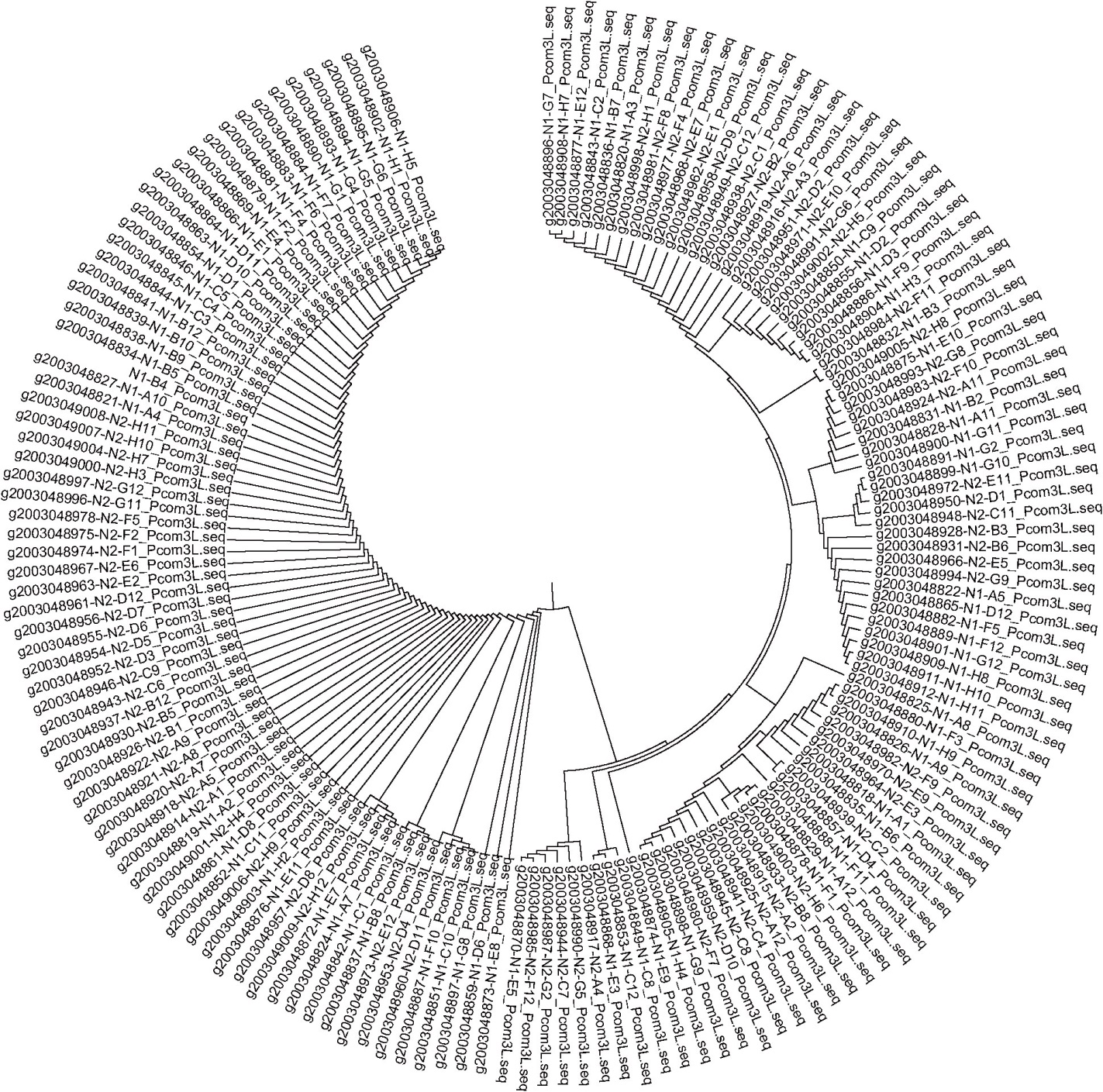


Figure S2. The circular form of the phylogenetic tree is based on variable regions sequences of kappa and lambda chains of selected Fabs. The phylogenetic trees were constructed by the minimum evolution method implemented of software MEGA6.

Figure S3


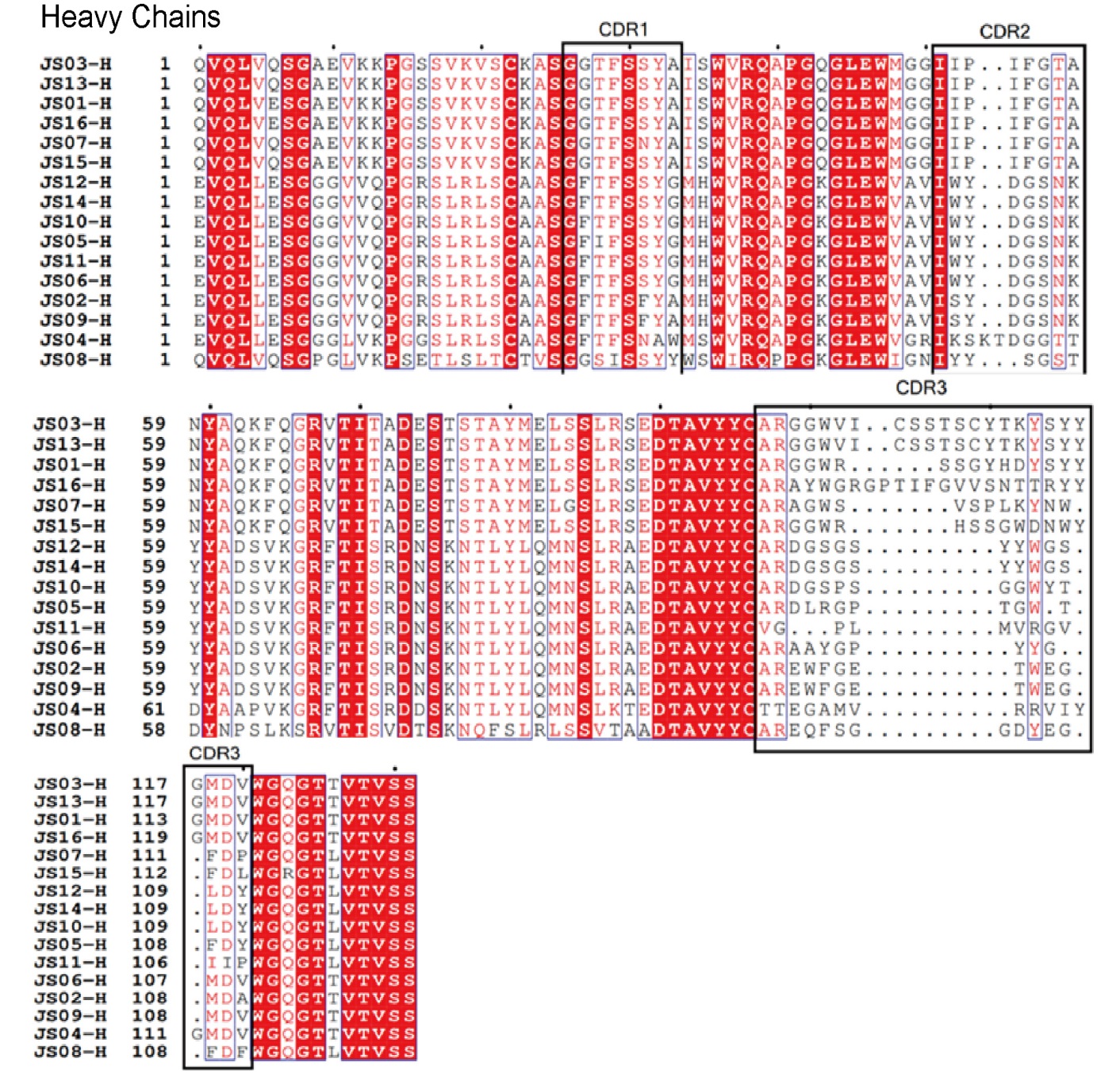


Figure S3. Sequence alignment for variable regions of heavy chains from 16 strains of SARS-CoV-2 NP-specific antibodies. The sequences were aligned using the web servers of Clustal Omega (<https://www.ebi.ac.uk/Tools/msa/clustalo>) and ESPript 3.0 (<http://espript.ibcp.fr/ESPript/ESPript>). CDRs of heavy chain were highlighted by black-bordered boxes.

Figure S4


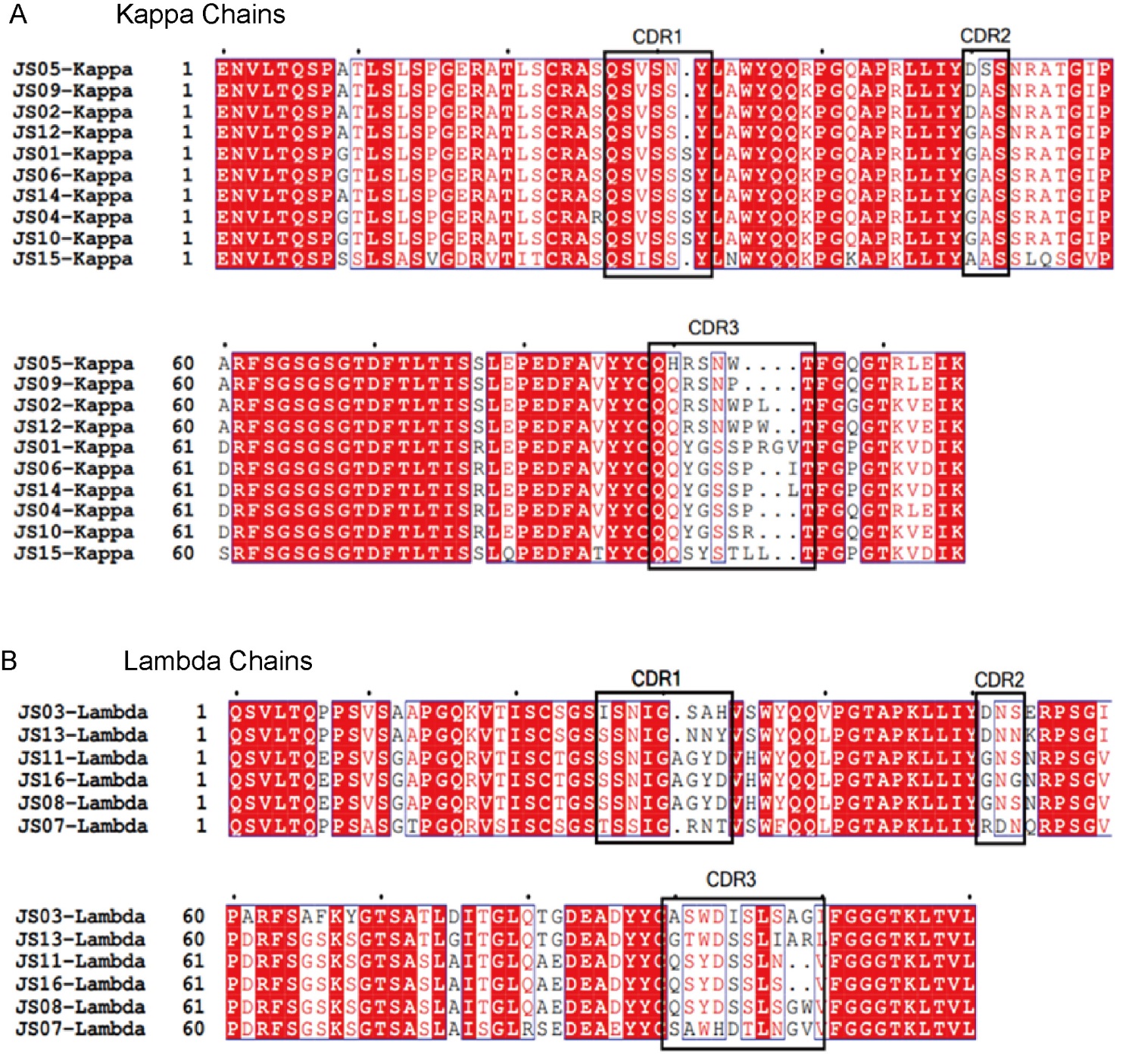


Figure S4. Sequence alignment for variable regions of kappa chains (A) and lambda chains (C) from 16 strains of SARS-CoV-2 NP-specific antibodies. Sequences were aligned using the web servers of Clustal Omega (<https://www.ebi.ac.uk/Tools/msa/clustalo>) and ESPript 3.0 (<http://espript.ibcp.fr/ESPript/ESPript>). CDRs of the heavy and light chain were highlighted by black-bordered boxes.

Figure S5


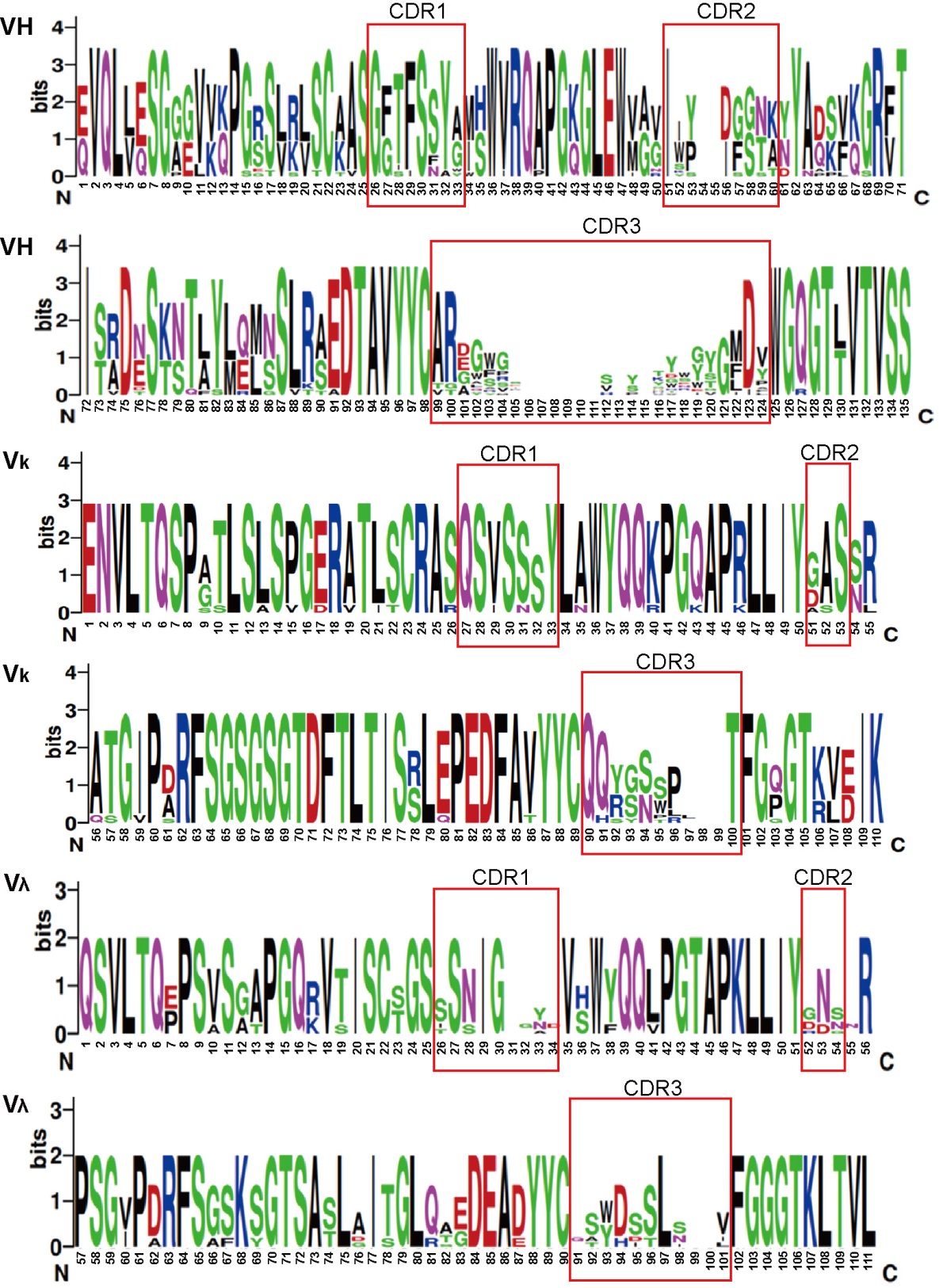


Figure S5. Multiple alignments of VH, Vκ, and Vλ of 16 antibodies generated by Weblogo (<http://weblogo.berkeley.edu/logo.cgi>). Logo plots revealed numerous differences in the variable regions. CDRs were highlighted in red boxes.

Figure S6


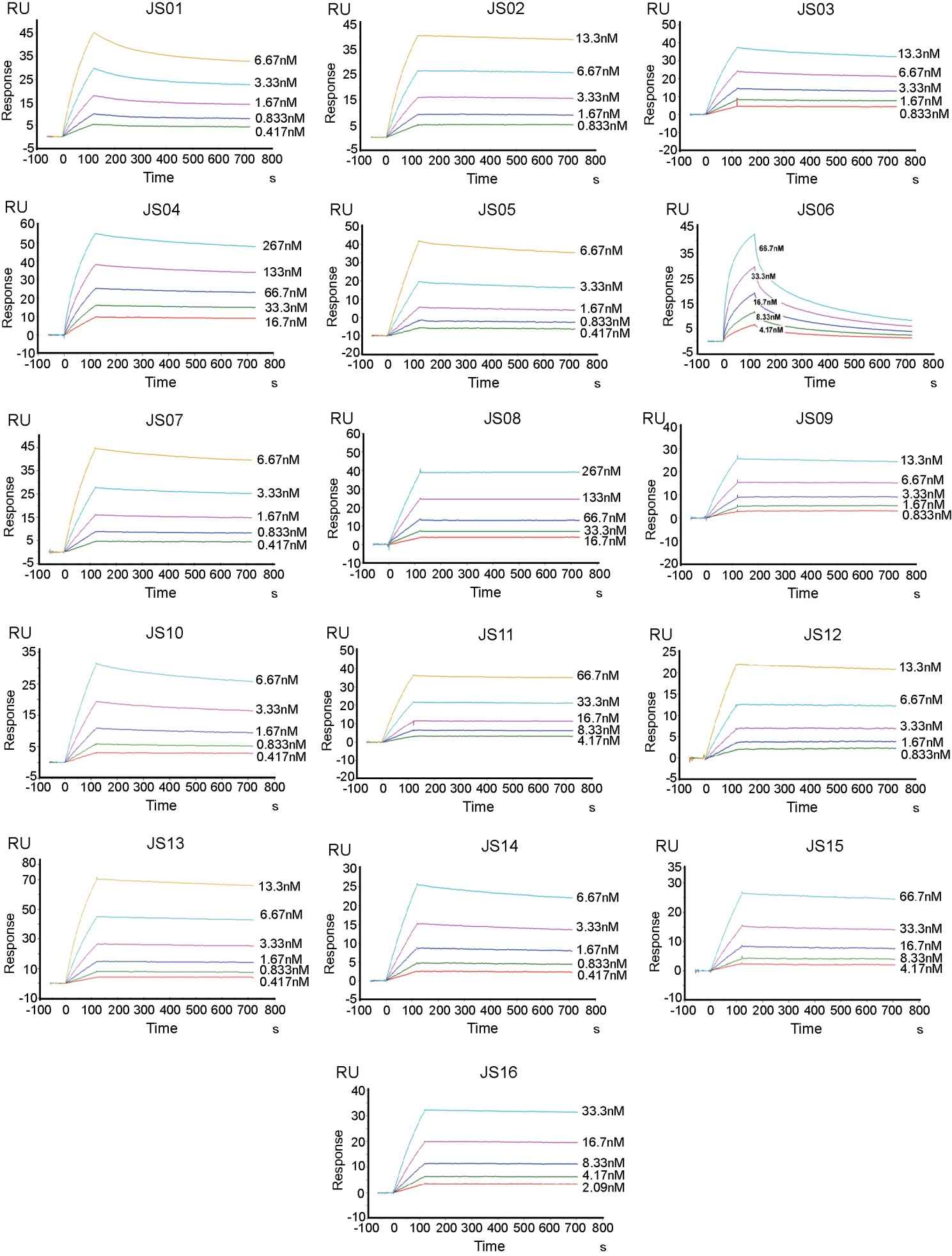


Figure S6. The kinetic curves of 16 antibodies against SARS-CoV-2 NP. SARS-CoV-2 NP was loaded on the CM5 sensor chip and tested for real-time association and dissociation of 16 human antibodies. Binding kinetics were evaluated using Biacore T200 Evaluation Software.

Figure S7


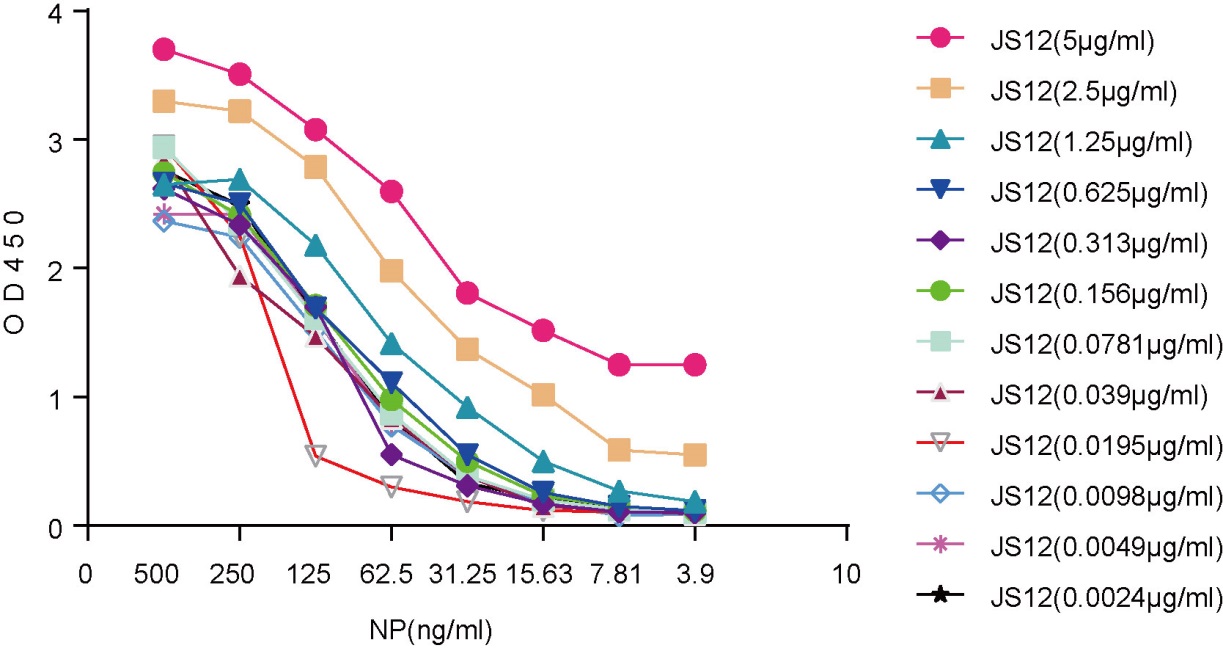


Figure S7. Evaluation of the optimal concentration of coated antibody and secondary antibody. The JS12 and JS08 were evaluated by the checkerboard. The horizontal axis shows different dilutions of SARS-CoV-2 NP. The different color lines mean different dilutions of the JS12 antibody.
